# The Nephroprotective Effects of Alpha-Mangostin for Acute Kidney Injury: A Systematic Review and Meta-Analysis

**DOI:** 10.3390/antiox14111374

**Published:** 2025-11-19

**Authors:** Moragot Chatatikun, Aman Tedasen, Ratana Netphakdee, Jitbanjong Tangpong, Phichayut Phinyo, Pakpoom Wongyikul, Fumitaka Kawakami, Makoto Kubo, Motoki Imai, Wiyada Kwanhian Klangbud, Atthaphong Phongphithakchai

**Affiliations:** 1 Department of Medical Technology, School of Allied Health Sciences, Walailak University, Nakhon Si Thammarat 80160, Thailand; moragot.ch@wu.ac.th (M.C.); aman.te@wu.ac.th (A.T.); ratana.ne@mail.wu.ac.th (R.N.); rjitbanj@wu.ac.th (J.T.); 2 Research Excellence Center for Innovation and Health Products (RECIHP), Walailak University, Nakhon Si Thammarat 80160, Thailand; 3 Center for Clinical Epidemiology and Clinical Statistics, Faculty of Medicine, Chiang Mai University, Chiang Mai 50200, Thailand; phichayut.phinyo@cmu.ac.th (P.P.); aumkidify@gmail.com (P.W.); 4 Department of Biomedical Informatics and Clinical Epidemiology (BioCE), Faculty of Medicine, Chiang Mai University, Chiang Mai 50200, Thailand; 5 Department of Regulation Biochemistry, Kitasato University Graduate School of Medical Sciences, Sagamihara 252-0373, Japan; kawakami@kitasato-u.ac.jp; 6 Department of Environmental Microbiology, Kitasato University Graduate School of Medical Sciences, Sagamihara 252-0373, Japan; kuboma@kitasato-u.ac.jp; 7 Regenerative Medicine and Cell Design Research Facility, School of Allied Health Sciences, Kitasato, Sagamihara 252-0373, Japan; imai-m@kitasato-u.ac.jp; 8 Department of Molecular Diagnostics, School of Allied Health Sciences, Kitasato University, 1-15-1 Kitasato, Sagamihara 252-0373, Japan; 9 Medical Technology Program, Faculty of Science, Nakhon Phanom University, Nakhon Phanom 48000, Thailand; wiyadakwanhian@gmail.com; 10 Nephrology Unit, Division of Internal Medicine, Faculty of Medicine, Prince of Songkla University, Hat Yai, Songkhla 90110, Thailand

**Keywords:** acute kidney injury, alpha-mangostin, creatinine, oxidative stress, anti-inflammatory

## Abstract

Acute kidney injury (AKI) is characterized by rapid loss of renal function due to oxidative stress, inflammation, and apoptosis, with limited targeted therapies. Alpha-mangostin (AM), a natural compound from *Garcinia mangostana*, exhibits antioxidant and anti-inflammatory properties in preclinical studies, but its efficacy in AKI has not been reviewed. This systematic review and meta-analysis, registered on the Open Science Framework and adhering to PRISMA guidelines, analyzed in vivo and in vitro studies on AM’s effects in AKI models through searches of PubMed, Scopus, Embase, and Web of Science. Primary outcomes included serum creatinine and cell viability, while secondary outcomes encompassed oxidative stress markers (malondialdehyde (MDA), glutathione (GSH), reactive oxygen species (ROS)), inflammatory cytokines, apoptosis indicators, and histopathology. Data were extracted independently and assessed using the Toxicological Data Reliability Assessment Tool (ToxRTool). AM significantly reduced serum creatinine (mean difference (MD) = −0.67 mg/dL; 95% confidence interval (CI): −1.28 to −0.06; *p* = 0.03) and improved cell viability (MD = 28.26%; 95% CI: 17.25 to 39.26; *p* < 0.0001). It markedly decreased MDA and ROS, increased GSH, and enhanced antioxidant enzymes (glutathione peroxidase (GPx), glutathione reductase (GR), superoxide dismutase (SOD)). In vivo, tumor necrosis factor-alpha (TNF-α) and interleukin-6 (IL-6) were lowered, and histopathology showed reduced tubular necrosis and structural damage. Subgroup analyses indicated dose- and model-dependent effects, with lower doses often yielding greater benefits. Sensitivity analyses confirmed robustness despite heterogeneity. Preclinical evidence supports AM’s nephroprotective potential and underscores the need for dose optimization, mechanistic validation, and clinical translation.

## 1. Introduction

Acute kidney injury (AKI) is a widespread condition marked by a sudden deterioration of kidney function, leading to the accumulation of waste substances such as serum creatinine and blood urea nitrogen (BUN), as well as fluid overload and electrolyte imbalance [1]. This abrupt decline in renal performance poses a significant health threat, especially among hospitalized and critically ill populations, due to its association with high rates of complications and mortality [2]. The pathogenesis of AKI is multifactorial, involving oxidative stress, inflammation, hypoxia, mitochondrial dysfunction, and apoptosis, which together lead to tubular and glomerular injury [3]. Recent mechanistic reviews have emphasized that oxidative stress-induced mitochondrial impairment, endothelial dysfunction, and maladaptive inflammatory signaling play central roles in the initiation and progression of AKI [4,5,6,7]. These studies also highlight the transition from acute tubular injury to chronic kidney disease through persistent oxidative and inflammatory cascades, reinforcing the need for therapies that target these molecular pathways. Despite the availability of supportive therapies like dialysis, targeted pharmacological options capable of preventing or reversing renal injury remain limited, highlighting the urgent need for innovative treatments aimed at preserving kidney function and improving patient outcomes [8].

AKI is a complex syndrome driven by oxidative stress, inflammation, and apoptosis, which collectively impair renal tubular integrity and function. Among the key molecular regulators, nuclear factor erythroid 2–related factor 2 (Nrf2) and nuclear factor kappa-light-chain-enhancer of activated B cells (NF-κB) play opposing roles in renal pathophysiology [9,10]. Nrf2 is a transcription factor that, upon activation, dissociates from its cytoplasmic inhibitor Keap1 and translocates to the nucleus, where it binds to antioxidant response elements (AREs) to induce genes encoding detoxifying and antioxidant enzymes such as heme oxygenase-1 (HO-1), NAD(P)H quinone oxidoreductase 1 (NQO1), glutathione peroxidase (GPx), glutathione reductase (GR), and superoxide dismutase (SOD), thereby maintaining redox homeostasis and protecting renal cells from oxidative injury [11]. Conversely, NF-κB is a pro-inflammatory transcription factor that, when activated by oxidative or nitrosative stress, undergoes nuclear translocation following inhibitor of NF-κB (IκB) degradation, leading to transcription of inflammatory mediators such as tumor necrosis factor—alpha (TNF-α) and interleukin-6 (IL-6), which are key drivers of renal inflammation and fibrosis [12]. Dysregulation of these pathways amplifies oxidative damage and inflammation in AKI. Natural compounds capable of activating Nrf2 while inhibiting NF-κB are increasingly recognized as promising therapeutic strategies.

Recent studies have increasingly highlighted the therapeutic potential of natural compounds in kidney disease, particularly due to their antioxidant, anti-inflammatory, and anti-apoptotic properties, which are critical for mitigating oxidative stress and inflammation in acute kidney injury (AKI) and renal fibrosis [13,14]. Natural products targeting the transforming growth factor β/Smad (TGF-β/Smad) signaling pathway have been shown to attenuate renal fibrosis by modulating extracellular matrix deposition and fibrotic signaling, as demonstrated in multi-omics-based reviews [15]. Emodin and rhein, the primary anthraquinones in *Rheum officinale*, provide renoprotective effects by reducing oxidative stress, suppressing inflammation, and inhibiting fibrotic signaling. These compounds lower ROS and MDA, enhance antioxidant enzymes such as SOD and GSH, downregulate pro-inflammatory cytokines (TNF-α, IL-6), and block TGF-β/Smad-mediated extracellular matrix deposition. Their combined antioxidant, anti-inflammatory, and anti-fibrotic actions contribute to improved renal function and structural preservation in AKI and chronic kidney disease (CKD) models [16]. Kaempferide, a natural flavonoid, demonstrates nephroprotective effects against cisplatin-induced AKI in both in vitro and in vivo models. Its mechanism involves inhibiting oxidative stress (reducing ROS and MDA, increasing SOD) and inducing autophagy (upregulating beclin-1, optineurin (OPTN), and microtubule-associated protein 1 light chain 3 (LC3 II), while modulating phosphorylated protein kinase B (p-AKT) and phosphorylated AMP-activated protein kinase (p-AMPK) [17]. Fisetin, a natural flavonoid, alleviates kidney injury in diabetes-exacerbated atherosclerosis by improving renal function and reducing morphological damage and fibrosis. Its effects are mediated through suppression of oxidative stress (ROS, advanced glycosylation end products (AGEs)) and inflammatory cytokines, inhibition of vascular endothelial growth factor A (VEGFA), fibronectin, and collagen expression, and activation of matrix metalloproteinases 2 and 9 (MMP2/MMP9). Mechanistically, fisetin inactivates TGF-β/Smad2/3 signaling and downregulates cluster of differentiation 36 (CD36), positioning it as a promising anti-fibrotic and nephroprotective agent [18]. Gut microbiota modulates renal fibrosis through its regulation of oxidative stress and inflammation, mediated by microbial metabolites and immune signaling. A balanced microbiota confers protective antioxidant and anti-inflammatory effects, whereas dysbiosis drives ROS production, cytokine activation, and fibrotic progression [19].

Alpha-mangostin (AM), a bioactive xanthone isolated from *Garcinia mangostana*, exhibits multiple pharmacological properties, including potent free radical scavenging [20,21], anti-inflammatory [22,23,24], antimicrobial [25,26,27,28], antiviral [29,30] and anticancer activities [31,32,33]. These properties make AM an attractive candidate for mitigating oxidative stress and inflammation underpinning kidney injury. Experimental models frequently employ nephrotoxic agents such as cisplatin, a widely used chemotherapeutic drug known to induce AKI, as well as glycerol, which is used to generate AKI models through rhabdomyolysis for evaluating protective interventions [34,35]. These drug- and toxin-induced models are instrumental in understanding how compounds like AM can prevent the decline in renal function associated with chemical injury [36]. However, all existing studies on AM have been limited to in vitro and animal models; no clinical studies or trials involving human subjects have been conducted to date, highlighting the significant gap in translating preclinical findings into human applications. Previous systematic reviews and meta-analyses have explored the antimicrobial activity and lipid-lowering effects of AM across various disease models and experimental settings [37,38]. Although these analyses indicate that AM has promising therapeutic potential, no comprehensive synthesis has been performed specifically regarding its efficacy in models of AKI. Given the central role of oxidative stress and inflammation in AKI pathogenesis, understanding the protective effects of AM in preclinical settings is essential to evaluate its potential as a candidate for future clinical trials. This systematic review and meta-analysis aimed to synthesize current preclinical evidence regarding the nephroprotective effects of AM in animal and cellular models of AKI.

## 2. Materials and Methods

### 2.1. Protocol and Registration

This systematic review and meta-analysis were registered with the Open Science Framework (OSF) prior to data extraction to promote transparency and reproducibility, following the Preferred Reporting Items for Systematic Reviews and Meta-Analyses (PRISMA) guidelines as shown in Table S1. The registration details, including the study protocol, search strategy, and planned analyses, are publicly available at https://doi.org/10.17605/OSF.IO/35SZN).

### 2.2. Literature Search

A systematic search was conducted to identify relevant studies evaluating the nephroprotective effects of AM in AKI. The search encompassed four major electronic databases—PubMed, Scopus, Embase, and Web of Science—up to 31 August 2025. The detailed search terms are provided in Supplementary Tables S2–S4. Only articles published in English were included. The search strategy employed specific combinations of keywords terms tailored to each database to ensure comprehensive retrieval of relevant literature.

### 2.3. Study Selection

The study selection process was conducted independently by three reviewers—M.C. and A.P.—in accordance with predefined inclusion and exclusion criteria to ensure the relevance and quality of the selected studies. Initially, titles and abstracts of all retrieved articles were screened independently by M.C. and A.P. to identify potentially eligible studies. Full texts of articles deemed relevant after the initial screening were subsequently obtained and thoroughly evaluated by all three reviewers (W.K.K.). Studies were included if they met the following criteria: (1) preclinical in vivo or in vitro experiments using animal models or renal cell lines pertinent to AKI; (2) intervention with purified AM administered alone; (3) reporting outcomes related to renal function (serum creatinine, blood urea nitrogen), oxidative stress, inflammatory cytokines, cell viability, or histopathological analysis; (4) inclusion of a control group receiving vehicle or placebo; and (5) full-text articles published in English.

Studies were excluded if they involved (1) human participants; (2) use of AM in combination with other agents; (3) lack of relevant outcomes such as renal function markers, oxidative stress, inflammatory cytokines, cell viability, or histopathology; (4) absence of a control group receiving vehicle or placebo; (5) non-English publications such as narrative reviews, editorials, conference abstracts, or other publications without accessible full text. Any disagreements between reviewers M.C. and A.P. were resolved through discussion or consultation with a third reviewer, S.S., to reach consensus. Table 1 presents the research question organized according to the PICOS (Population, Intervention, Comparison, Outcomes, and Study) framework.

### 2.4. Data Extraction

The data extraction process involved systematically collecting relevant information from each included study. Specifically, data were extracted on the study characteristics (authors, publication year, animal model or cell line), details of the intervention (dose, duration of AM administration), type of comparator used, methods of AKI induction, measured outcomes (such as serum creatinine, BUN, oxidative stress markers, inflammatory cytokines, apoptosis indicators, histopathological findings), and key findings related to the effects of AM. M.C. and A.P. performed the data extraction, and W.K.K. served as the third reviewer. Discrepancies were resolved through discussion or consultation with W.K.K. to ensure accuracy and consistency. The units of BUN and creatinine were recorded in mg/dL, and cell viability was expressed as a percentage. Continuous outcomes, expressed as mean ± standard deviation (SD), were imputed following the guidelines outlined in the Cochrane Handbook for Systematic Reviews of Interventions, and then converted to standard errors of the mean (SEMs) using the formula: SEM is obtained by dividing the SD by the square root of the sample size (*n*) [39]. If necessary, additional data were obtained by contacting the study authors through email. The primary outcomes included serum creatinine, BUN, and cell viability, while secondary outcomes encompassed oxidative stress markers, inflammatory cytokines, apoptosis indicators, and histopathological findings.

### 2.5. Quality Assessment of Included Studies

The quality assessment of the included in vitro and in vivo studies was conducted using the Toxicological Data Reliability Assessment Tool (ToxRTool), a standardized instrument for evaluating the reliability of toxicological data [40]. The ToxRTool assesses various criteria related to data quality. Each criterion was scored as 1 (“criterion met”) or 0 (“criterion not met”). The assessment evaluated key aspects including (i) identification of the test substance, (ii) characterization of the test system, (iii) description of the study design, (iv) documentation of study results, and (v) the plausibility of both the study design and findings. The total score was 18 for the in vitro studies and 21 for the in vivo studies. Additionally, reliability categorization was conducted according to Klimisch et al., as follows: in vivo studies scored 18–21 points (category I) and were considered reliable without restrictions; 13–17 points (category II) and were considered reliable with restrictions; less than 13 points or not meeting all red criteria were deemed not reliable (category III). For in vitro studies, scores of 15–18 points (category I) indicated reliability without restrictions; 11–14 points (category II) indicated reliability with restrictions; and below 11 or not meeting all criteria (category III) were considered not reliable [41]. The assessment was independently performed by two reviewers (M.C. and A.P.), and discrepancies were resolved through discussion or consultation with a third reviewer (W.K.K.) to ensure consistent and accurate quality evaluation.

### 2.6. Data Synthesis and Statistical Analysis

Data synthesis and statistical analysis were performed using STATA version 17 (StataCorp LLC, College Station, TX, USA). A random-effects model based on the DerSimonian–Laird method was applied to account for between-study variability. For primary outcomes such as serum creatinine, blood urea nitrogen (BUN), and cell viability, pooled estimates were expressed as mean differences (MD) with 95% confidence intervals (CI). For secondary outcomes (oxidative stress markers, inflammatory cytokines, apoptosis indicators), which were reported in different units across studies, standardized mean differences (SMD) were calculated using Hedges’ g to ensure comparability [42].

Statistical heterogeneity was assessed using Cochran’s Q test and quantified by the *I*^2^ statistic, interpreted as low (0–25%), moderate (26–50%), substantial (51–75%), or considerable (>75%) [43]. When heterogeneity exceeded 50%, subgroup analyses were conducted where sufficient data were available [38]. Specifically, subgroup analysis was performed for in vitro studies based on cell type (LLC-PK1 vs. HEK293), while subgroup analysis for in vivo studies was not possible due to the limited number of included studies (*n* = 2). Additional subgroup factors considered included AKI induction model, route of AM administration, and dose categories when data permitted.

Sensitivity analyses were performed by recalculating pooled estimates using the average, lowest, and highest doses reported across studies to evaluate the robustness of findings and explore dose–response relationships. This approach assesses the robustness of the results and the potential impact of variability in creatinine measurements. Publication bias was planned to be assessed using funnel plots and Egger’s regression test for outcomes with at least 10 studies; however, this was not performed due to the limited number of included studies. Statistical significance was determined using *Z*-tests, with a two-tailed *p*-value < 0.05 considered significant [44]. Forest plots were generated to visually present individual study effects and overall pooled estimates.

## 3. Results

### 3.1. Search Outcomes

**Figure 1:**
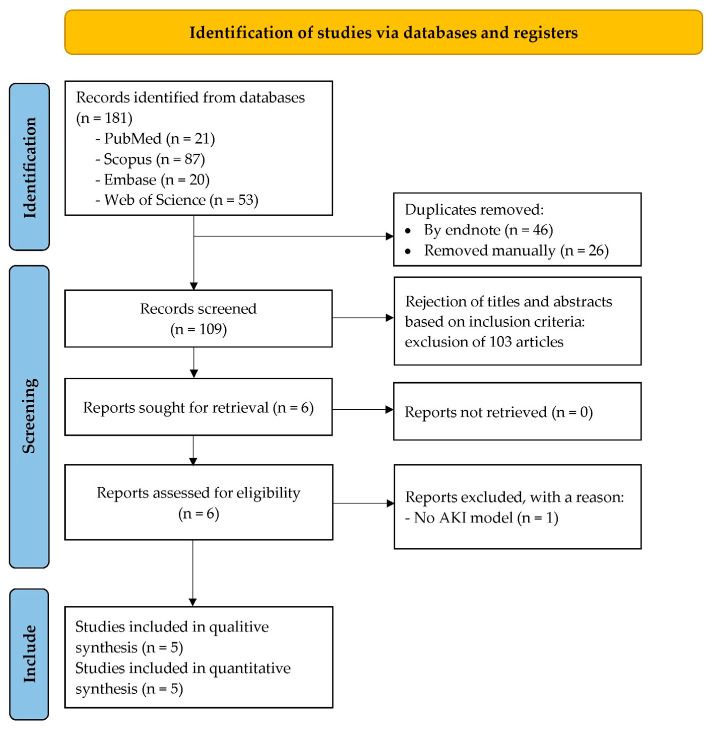
PRISMA flow diagram showing the selection process of studies included in the review.

The study selection process is summarized in Figure 1. Initially, 181 records were identified from databases and registers, including PubMed (*n* = 21), Scopus (*n* = 87), Embase (*n* = 20), and Web of Science (*n* = 53). After removing duplicates (*n* = 72), a total of 109 unique records were screened based on titles and abstracts, leading to the exclusion of 103 articles that did not meet the inclusion criteria. The full texts of 6 reports were then assessed for eligibility, resulting in the exclusion of 1 report due to the absence of an AKI model. The remaining five studies satisfied the eligibility criteria and were included in the review. These studies were incorporated into both the qualitative and quantitative syntheses.

### 3.2. Study Characteristics

Table 2 summarizes the characteristics of the five studies included in the qualitative and quantitative syntheses. The studies were conducted in Egypt (*n* = 1) [45], the United States (*n* = 3) [46,47,48], and China (*n* = 1) [49] and employed both in vivo (*n* = 2) [45,46] and in vitro (*n* = 3) [47,48,49] models of AKI. The animal models utilized male Sprague Dawley (*n* = 1) [45] or Wistar rats (*n* = 1) [46], while in vitro studies employed LLC-PK1 renal epithelial cells (*n* = 2) [47,48] or HEK293 epithelial cells (*n* = 1) [49]. AKI was induced through glycerol (*n* = 1) [45] or cisplatin (*n* = 4) [46,47,48,49] administration. Sample sizes per group ranged from 3 to 10 subjects/samples. The duration of AM administration varied from 1 day (*n* = 3) [47,48,49] to 3 days (*n* = 1) [45] to 6–10 days (*n* = 1) [46], with doses ranging from 12.5 mg/kg to 200 mg/kg for in vivo studies and 1 μM to 40 μM for in vitro studies. In vivo control groups primarily consisted of animals treated with glycerol (*n* = 1) [45] or with cisplatin and saline (n = 1) [46]. In contrast, in vitro control groups were treated only with cisplatin (*n* = 3) [47,48,49]. Primary outcomes assessed included serum creatinine (*n* = 2) [45,46], BUN (*n* = 1) [46], and cell viability (*n* = 3) [47,48,49], while secondary outcomes consisted of oxidative stress markers.

### 3.3. Quality Assessment of Included Studies

The quality assessment of five studies, two in vivo and three in vitro, was assessed using the ToxRTool, which evaluates five key criteria as shown in Table 3. For the in vivo studies, Eltahir et al., 2023 scored 18 and Pérez-Rojas et al. scored 20; both were classified as reliable without restrictions [45,46]. Among the in vitro studies, Sánchez-Pérez et al. 2010 scored 18, while Reyes-Fermín et al., 2019 and Li et al., 2020 scored 17; all were classified as reliable without restrictions [47,48,49]. These results support the inclusion of all five studies in the review, with no restrictions on reliability classification.

### 3.4. Effect of AM on Primary Outcomes

#### 3.4.1. Effect of AM on Serum Creatinine Levels in In Vivo Models of AKI

**Figure 2:**
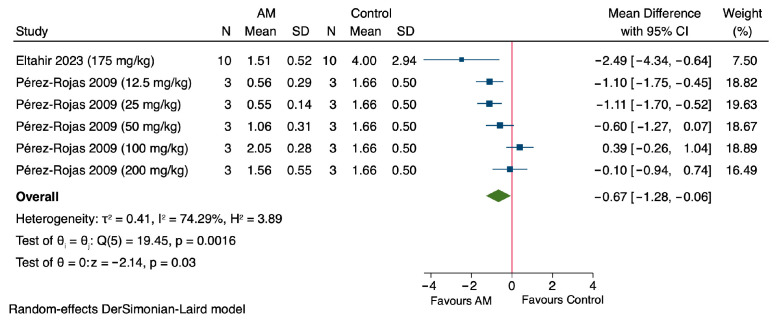
Forest plot illustrating the effect of alpha-mangostin (AM) on serum creatinine levels in in vivo models of acute kidney injury (AKI) [45,46].

The meta-analysis of the included studies indicates that AM significantly reduces serum creatinine levels in experimental models of acute kidney injury as shown in Figure 2. Specifically, Eltahir et al., 2023, which employed glycerol to induce AKI, demonstrated a substantial decrease with a MD of −2.49 mg/dL (95% CI: −4.34 to −0.64) at a dose of 175 mg/kg [45]. Pérez-Rojas et al. (2009), which used cisplatin to induce AKI, showed a dose-dependent reduction in serum creatinine levels: at 12.5 mg/kg, the MD was −1.10 mg/dL (95% CI: −1.75 to −0.45); at 25 mg/kg, −1.11 mg/dL (95% CI: −1.70 to −0.52); at 50 mg/kg, −0.60 mg/dL (95% CI: −1.27 to 0.07); at 100 mg/kg, 0.39 mg/dL (95% CI: −0.26 to 1.04); and at 200 mg/kg, −0.10 mg/dL (95% CI: −0.94 to 0.74) [46]. The overall pooled estimate across these studies revealed a significant effect favoring AM, with a MD of −0.67 mg/dL (95% CI: −1.28 to −0.06). The Z-value was −2.14 (*p* = 0.03), indicating statistical significance. The heterogeneity among studies was substantial (τ^2^ = 0.41, *I*^2^ = 74.29%), and Cochran’s Q test yielded a Q-value of 19.45 with a *p* value of 0.0016, confirming significant heterogeneity. Despite this variability, the findings support the nephroprotective effect of AM in reducing serum creatinine levels in preclinical AKI models.

#### 3.4.2. Effect of AM on Cell Viability in In Vitro Models of Cisplatin-Induced Nephrotoxicity

**Figure 3:**
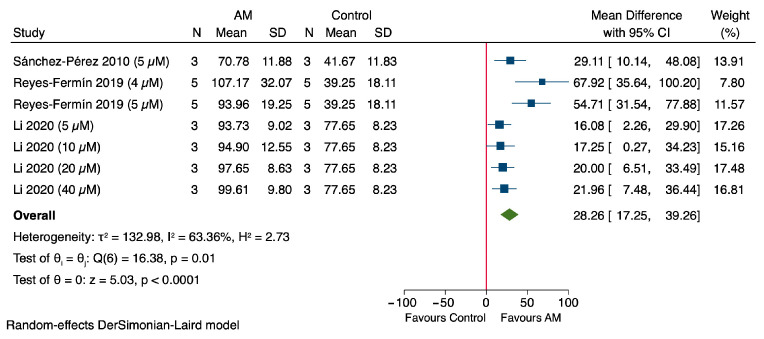
Forest plot illustrating the effect of alpha-mangostin (AM) on cell viability in in vitro models of cisplatin-induced nephrotoxicity [47,48,49].

The results of the meta-analysis demonstrate that AM significantly enhances cell viability, expressed as a percentage, in in vitro models of cisplatin-induced nephrotoxicity, with treatment administered for one day across all included studies as shown in Figure 3. Notably, Sánchez-Pérez et al., 2010, using a concentration of 5 μM, reported a MD of 29.11% (95% CI: 10.14 to 48.08) in cell viability compared to the control group, using LLC-PK1 renal epithelial cells [47]. Reyes-Fermín et al. (2019), at 4 μM of AM, exhibited a MD of 67.92% (95% CI: 35.64 to 100.20), also using LLC-PK1 cells [48]. Furthermore, Reyes-Fermín et al. (2019) at 5 μM, as well as Li et al. (2020) at concentrations of 5, 10, 20, and 40 μM, demonstrated MDs of 54.71% (95% CI: 31.54 to 77.88), 16.08% (95% CI: 2.26 to 29.90), 17.25% (95% CI: 0.27 to 34.23), 20.00% (95% CI: 6.51 to 33.49),and 21.96% (95% CI: 7.48 to 36.44), respectively, using HEK293 epithelial cells [49]. The overall pooled estimate revealed a significant increase in cell viability, with a MD of 28.26% (95% CI: 17.25 to 39.26). The heterogeneity among the included studies was considerable (τ^2^ = 132.98, *I*^2^ = 63.36%), and Cochran’s Q test yielded a value of 16.38 (*p* = 0.01). The overall effect was statistically significant, with a Z value of −5.03 and a *p* value < 0.00001, indicating a highly significant protective effect of AM on cell viability in cisplatin-induced nephrotoxicity models.

### 3.5. Effect of AM on Secondary Outcomes

#### 3.5.1. Effect of AM on MDA Levels in In Vivo and In Vitro Models

**Figure 4:**
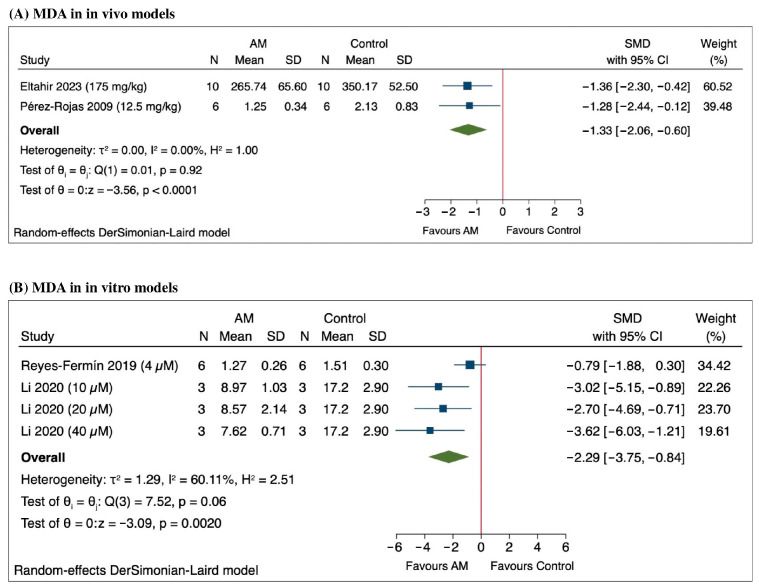
Forest plot of the standardized mean differences (SMD) in malondialdehyde (MDA) levels following alpha-mangostin (AM) treatment in (**A**) in vivo models [45,46] and (**B**) in vitro models [48,49].

The meta-analysis assessing MDA levels demonstrates a significant reduction in oxidative stress following treatment with AM as shown in Figure 4A. Specifically, Eltahir et al., 2023 reported a SMD of −1.36 (95% CI: −2.30 to −0.42), while Pérez-Rojas et al., 2009 observed an SMD of −1.28 (95% CI: −2.44 to −0.12) (6, 7). The pooled estimate across studies revealed an SMD of −1.33 (95% CI: −2.06 to −0.60), indicating a protective effect of AM against oxidative stress [45,46]. Heterogeneity was low (τ^2^ = 0.00, *I*^2^ = 0.00%), suggesting consistency among the included studies. The overall effect was statistically significant (Z = −3.56, *p* < 0.0001), supporting the potential role of AM in reducing MDA in animal models of acute kidney injury.

The meta-analysis of the in vitro studies measuring MDA levels following treatment with AM revealed a significant overall reduction, with a SMD of −2.29 (95% CI: −3.75 to −0.84) as shown in Figure 4B. Notably, Reyes-Fermín et al. (2019) reported an SMD of −0.79 (95% CI: −1.88 to 0.30), indicating a trend toward decreased MDA levels, although this was not statistically significant [48]. Li et al. (2020) demonstrated significant reductions at different concentrations: an SMD of −3.02 (95% CI: −5.15 to −0.89) at 10 μM, −2.70 (95% CI: −4.69 to −0.71) at 20 μM, and −3.62 (95% CI: −6.03 to −1.21) at 40 μM [49]. Heterogeneity among the studies was substantial (τ^2^ = 1.29, *I*^2^ = 60.11%), and the overall effect was statistically significant, with a Z value of −3.09 (*p* = 0.0020), based on a random-effects DerSimonian-Laird model. These results support a consistent decrease in MDA levels following AM treatment across the included in vitro studies.

#### 3.5.2. Effect of AM on Glutathione (GSH) Levels in In Vitro Models

**Figure 5:**
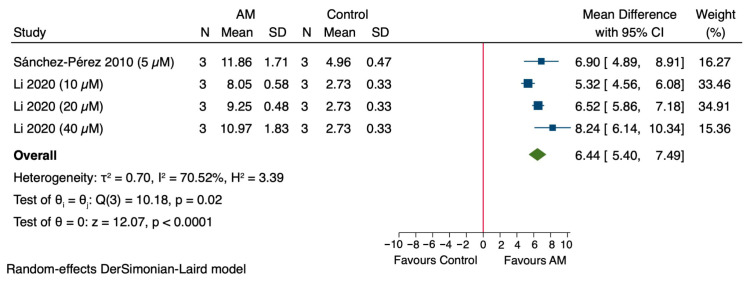
Forest plot illustrating the effect of alpha-mangostin (AM) on glutathione (GSH) levels in in vitro models [47,49].

The pooled analysis of the studies assessing the effect of AM on GSH levels in in vivo models revealed a significant overall increase, with an MD of 6.44 (95% CI: 5.40 to 7.49), as shown in Figure 5. Individual studies showed varying effects: Sánchez-Pérez et al., 2010 reported an MD of 6.90 (95% CI: 4.89 to 8.91), Li et al., 2020 at different concentrations demonstrated increases with MDs of 5.32 (95% CI: 4.56 to 6.08) at 10 μM, 6.52 (95% CI: 5.86 to 7.18) at 20 μM, and 8.24 (95% CI: 6.14 to 10.34) at 40 μM [47,49]. Heterogeneity among the studies was substantial (τ^2^ = 0.70, *I*^2^ = 70.52%, H^2^ = 3.39), and the overall effect was statistically significant (Z = 12.07, *p* < 0.0001). These results suggest that treatment with AM significantly increases GSH levels in in vitro models.

#### 3.5.3. Effect of AM on Reactive Oxygen Species (ROS) Levels in In Vitro Models

**Figure 6:**
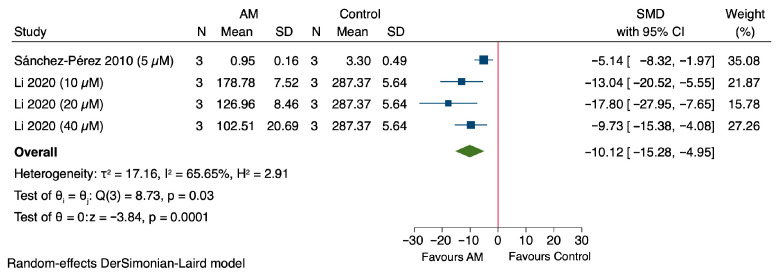
Forest plot illustrating the effect of alpha-mangostin (AM) on reactive oxygen species (ROS) levels in in vitro models [47,49].

The meta-analysis demonstrated that treatment with AM significantly decreased ROS levels in cells, with a pooled SMD of −10.12 (95% CI: −15.28 to −4.95) as shown in Figure 6. Individual studies showed reductions: Sánchez-Pérez et al., 2010 reported an SMD of −5.14 (95% CI: −8.32 to −1.97), Li et al., 2020 at different concentrations demonstrated SMDs of −13.04 (95% CI: −20.52 to −5.55) at 10 μM, −17.80 (95% CI: −27.95 to −7.65) at 20 μM, and −9.73 (95% CI: −15.38 to −4.08) at 40 μM [47,49]. Heterogeneity among the studies was substantial (τ^2^ = 17.16, *I*^2^ = 65.65%, H^2^ = 2.91), and the overall effect was statistically significant (Z = −3.84, *p* < 0.0001). These results indicate that AM significantly reduces ROS levels in cells.

### 3.6. Nephroprotective Effects in AKI Models

In in vivo models of kidney injury, AM treatment was associated with notable changes in both oxidative stress and inflammatory markers as shown in Table 4. Specifically, there was an observed increase in GPx, GRs, and SOD levels [45]. Additionally, AM led to a reduction in renal hydrogen peroxide (H_2_O_2_), protein carbonyls, 4-HNE, and 3-NT [46]. Furthermore, it decreased the levels of the inflammatory markers (TNF-α and IL-6) [45]. These combined findings suggest that AM promotes an enhanced antioxidant defense system, reduces oxidative and nitrosative damage, and exerts anti-inflammatory effects within kidney tissues in vivo.

In both studies, AM demonstrated significant histopathological protection in models of AKI as shown in Table 4. In the glycerol-induced rhabdomyolysis model by Eltahir et al., 2023, untreated AKI rats showed severe degeneration of renal corpuscles, tubular necrosis, and protein cast accumulation [45]. AM treatment markedly alleviated these changes, restoring normal renal corpuscles and tubules with minimal degeneration and reduced protein deposition [45]. Similarly, in the cisplatin-induced nephrotoxicity model by Pérez-Rojas et al., 2009, rats treated with cisplatin exhibited extensive renal damage, including necrosis, vacuolization, and hyaline cast formation, affecting approximately 30% of the proximal tubular area [46]. However, co-treatment with AM reduced the damaged area to 11%, preserving renal architecture and minimizing structural injury [46]. These findings confirm AM’s renoprotective effects at the structural level in different AKI models.

AM effectively mitigates cisplatin-induced renal cell apoptosis by targeting multiple apoptotic pathways as summarized in Table 4. In LLC-PK1 cells, AM reduced oxidative stress and suppressed p53 upregulation, thereby decreasing apoptosis [47]. In HEK293 cells, AM inhibited the activation of caspase-3, caspase-9, and prevented poly (ADP-ribose) polymerase (PARP) cleavage, while restoring the balance of B-cell lymphoma 2 (Bcl-2) family proteins—reducing pro-apoptotic Bcl-2-associated X protein (Bax) and Bcl-2-associated death promoter (Bad), and increasing anti-apoptotic Bcl-2 and Bcl-2 extra-large (Bcl-xl) [49]. These findings highlight AM’s antioxidant and anti-apoptotic properties through modulation of tumor suppressor protein p53 and caspase cascades apoptotic regulators.

The in vitro findings strongly supported the in vivo observations as shown in Figure 2 and Figure 3, and Table 4. AM enhanced cell viability in cisplatin-induced nephrotoxicity models and reduced oxidative stress markers such as MDA and ROS, while increasing glutathione GSH levels. These molecular changes were accompanied by suppression of apoptotic signaling pathways, including inhibition of p53, caspase-3/9, and PARP cleavage, and restoration of Bcl-2 family balance. These effects align with in vivo results, where AM lowered serum creatinine, improved antioxidant enzyme activity (GPx, GR, and SOD), and reduced renal oxidative/nitrosative stress markers such as H_2_O_2_, protein carbonyls, 4-HNE, and 3-NT. Additionally, AM decreased inflammatory cytokines (TNF-α, IL-6) and preserved renal histology by minimizing tubular necrosis and structural damage. Collectively, the cellular-level improvements in viability and oxidative/apoptotic regulation provide mechanistic support for the functional and structural renoprotection observed in animal models.

### 3.7. Subgroup Analysis

**Figure 7:**
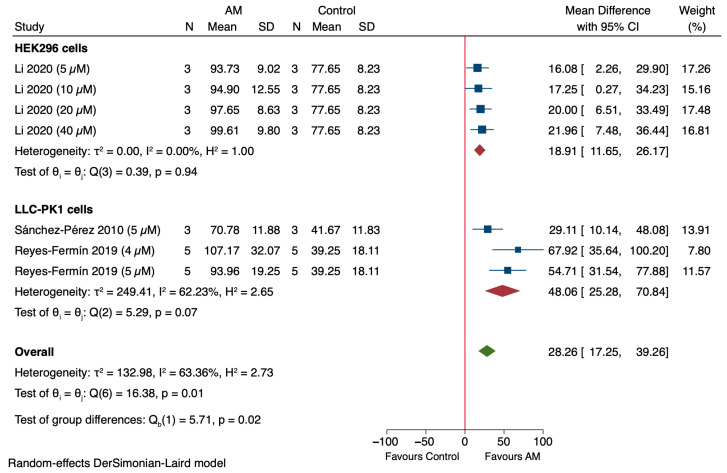
Subgroup analysis of the effect of alpha-mangostin (AM) on cell viability in in vitro models [47,48,49].

Subgroup analysis for in vivo studies was not performed due to the limited number of available studies (*n* = 2), which is insufficient for reliable statistical comparison [45,46]. The subgroup analysis evaluated the effect of alpha-mangostin (AM) on cell viability in HEK296 and LLC-PK1 cell models [47,48,49]. In HEK296 cells, a single study (Li et al., 2020) reported consistent increases in cell viability across concentrations of 5–40 µM, with a pooled MD of 18.91% (95% CI: 11.65 to 26.17) and low heterogeneity (*I*^2^ = 0.00%), as shown in Figure 7 [49]. In LLC-PK1 cells, two studies showed a positive effect of AM with a pooled MD of 48.06% (95% CI: 25.28 to 70.84), though moderate heterogeneity was observed (*I*^2^ = 62.23%), as shown in Figure 7 [47,48]. These subgroup analyses showed no significant difference within each subgroup, with *p*-values of 0.94 and 0.07, respectively. A test for subgroup differences indicated a statistically significant variation between cell types (*p* = 0.02), suggesting cell-specific responses to AM.

### 3.8. Sensitivity Analysis

**Figure 8:**
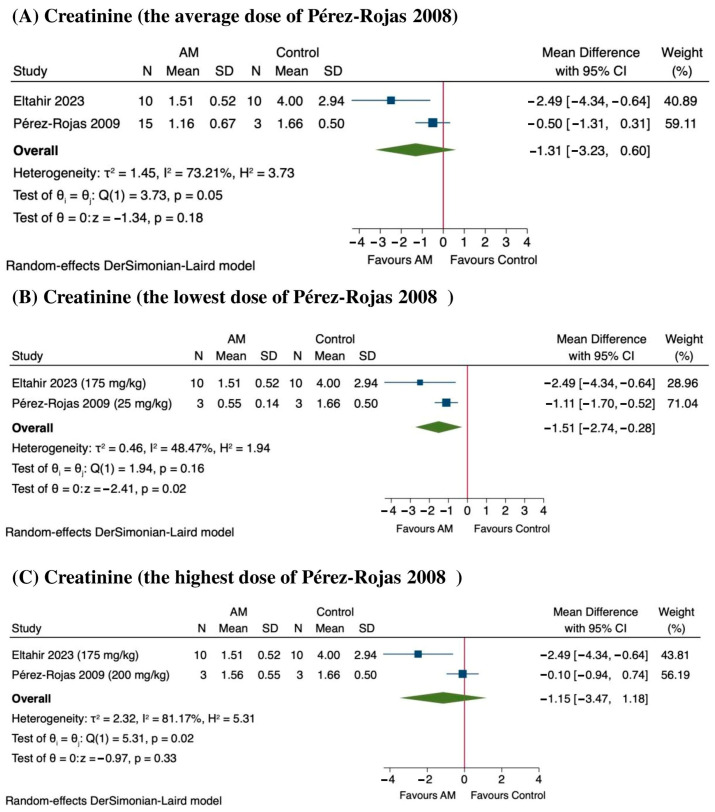
Sensitivity analysis of the effect of alpha-mangostin (AM) on serum creatinine levels at different dose ranges [45,46]. (**A**) Effect at the average dose, (**B**) effect at the lowest dose, and (**C**) effect at the highest dose.

The sensitivity analysis results indicate that AM tends to reduce creatinine levels, suggesting potential nephroprotection, although the outcomes vary depending on the dose [45,46]. When considering the average values from Pérez-Rojas et al., 2009, there was a MD of −1.31 mg/dL (95% CI: −3.23 to 0.60), with a *p* value of 0.18, and substantial heterogeneity across studies (*I*^2^ = 73.21%) as shown in Figure 8A. At the lowest dose reported in Pérez-Rojas et al., 2009 (25 mg/kg of AM), creatinine levels significantly decreased by an MD of −1.51 mg/dL (95% CI: −2.74 to −0.28), with a *p*-value of 0.02 and moderate heterogeneity (*I*^2^ = 48.47%) as shown in Figure 8B. Conversely, at the highest dose of 200 mg/kg, the MD was −1.15 mg/dL (95% CI: −3.47 to 1.18), which was also not statistically significant (*p* = 0.33), with considerable heterogeneity (*I*^2^ = 81.17%) as shown in Figure 8C. The sensitivity analysis suggested that lower doses of the treatment may be more effective in reducing creatinine levels, while higher doses do not confer additional benefit and may introduce variability. These findings support dose optimization in future studies.

**Figure 9:**
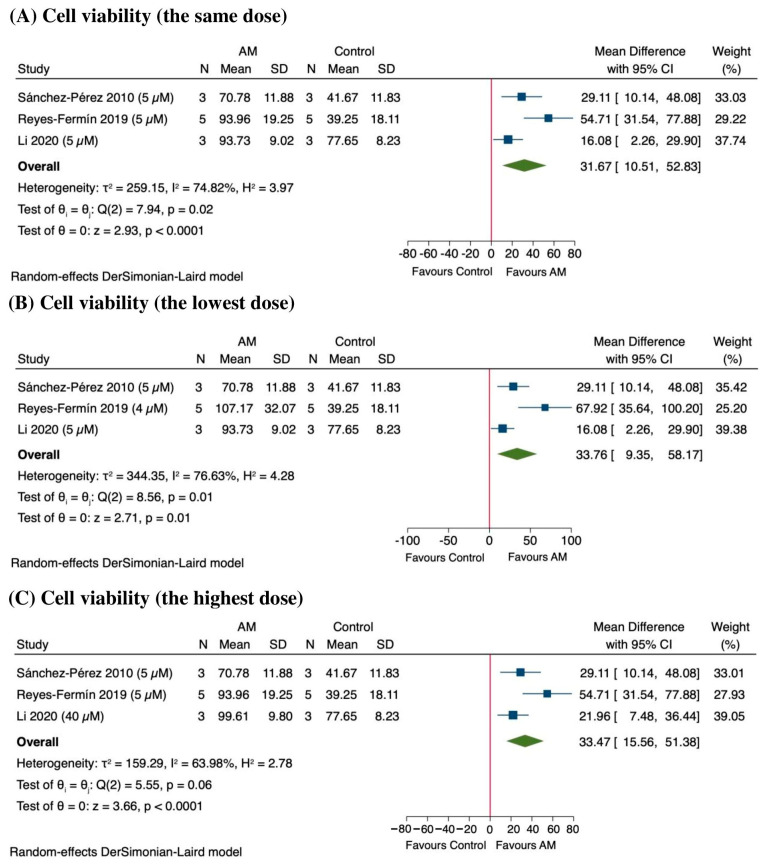
Sensitivity analysis of the effect of alpha-mangostin (AM) on cell viability at different doses [47,48,49]. (**A**) Effect at the same dose across studies, (**B**) effect at the lowest dose, and (**C**) effect at the highest dose.

In the sensitivity analysis, the results indicate that AM significantly enhances cell viability across different doses, particularly in studies by Reyes-Fermín et al., 2019, and Li et al., 2020 [48,49]. At the same dose (5 µM), the pooled MD in cell viability was approximately 31.67%, (95% CI: 10.51 to 52.83; *p* < 0.0001), indicating a statistically significant improvement as shown in Figure 9A. However, there was substantial heterogeneity among the included studies (*I*^2^ = 74.82%), suggesting variability in treatment response. At the lowest dose, the effect remained significant, with a MD of 33.76% (95% CI: 9.35 to 58.17; *p* = 0.01), although heterogeneity remained considerable (*I*^2^ = 76.63%) as shown in Figure 9B. Similarly, at the highest dose, AM treatment resulted in an increase of 33.47% (95% CI: 15.56 to 51.38; *p* < 0.0001), with substantial heterogeneity (*I*^2^ = 63.98%) as shown in Figure 9C. These findings suggest a consistent dose-dependent enhancement of cell viability following AM treatment, although the observed heterogeneity warrants further investigation into potential moderating factors.

### 3.9. Publication Bias

Publication bias was not formally assessed for either the in vivo (*n* = 2) or in vitro (*n* = 3) subgroups due to the limited number of included studies. Funnel plot asymmetry tests, such as Egger’s regression, generally require a minimum of 10 studies to generate reliable results. With fewer studies, these tests are underpowered and may produce misleading conclusions. Consequently, a definitive evaluation of publication bias was not conducted in this analysis.

## 4. Discussion

This systematic review and meta-analysis, synthesizing evidence from five preclinical investigations employing both in vivo (glycerol- and cisplatin-induced AKI models in rats) [45,46] and in vitro (cisplatin-induced toxicity in LLC-PK1 and HEK293 renal epithelial cells) models [47,48,49], provides compelling support for the nephroprotective effects of AM. Across both in vivo and in vitro studies, AM consistently demonstrated beneficial effects on renal function, cell viability, oxidative stress markers, inflammatory markers, apoptosis markers, and histopathological outcomes, suggesting its potential as a therapeutic agent in AKI management. The rigorous quality assessment, employing ToxRTool and yielding a categorization of all included studies as reliable without restrictions (Category I), enhances the confidence in the validity and robustness of the findings presented in this meta-analysis.

Regarding serum creatinine, a key biomarker of renal function, our meta-analysis reveals a statistically significant reduction following AM administration across in both glycerol- and cisplatin-induced AKI models, indicating its renoprotective potential [45,46]. Similarly, Santoso et al. (2022) also confirmed the creatinine-lowering effects of AM in a rat model of type 2 diabetes [50]. Furthermore, AM also showed a significant elevation in creatinine clearance within insulin resistance rat model, further supporting its role in metabolic kidney injury [51]. When compared to other natural compounds such as curcumin and resveratrol, Li et al. (2018) reported that curcumin administration significantly lowered serum creatinine levels in a rat model of cisplatin-induced nephrotoxicity, an effect attributed to its anti-inflammatory and antioxidant properties [52]. Consistent with our findings, research on resveratrol by Wang et al. (2019) demonstrated that this polyphenol could reduce creatinine levels and improve renal function in a model of ischemia–reperfusion injury [53]. The observed reductions in creatinine align with a broader pattern of renoprotection, lending support to the potential therapeutic utility of AM in mitigating kidney injury. These findings collectively reinforce AM’s therapeutic promise in renal injury, though clinical validation remains essential.

In addition to the observed improvements in serum creatinine, the findings of this meta-analysis indicate that AM demonstrably enhances cell viability under conditions of nephrotoxic injury, particularly in renal epithelial cells exposed to cisplatin, supporting its cytoprotective role in acute kidney injury (AKI) [47,48,49]. This effect is attributed to its ability to preserve mitochondrial function, reduce oxidative stress, and inhibit apoptosis [48,49]. Reyes-Fermín et al. demonstrated that AM prevented mitochondrial fragmentation and mitophagy in LLC-PK1 cells, while Li et al. reported activation of phosphatidylinositol 3-kinase (PI3K)/Protein Kinase B (Akt) and suppression of c-Jun N-terminal kinase (JNK) signaling in HEK293 cells [48,49]. Notably, AM has also been shown to improved cell viability on 1-methyl-4-phenylpyridinium (MPP+)-induced apoptotic cell death in neuroblastoma SH-SY5Y cells [54]. Furthermore, other compounds, such as N-acetylcysteine (NAC), a well-established antioxidant, has demonstrated the ability to significantly improve cell viability in renal tubular cells exposed to cisplatin [55]. Similarly, studies on epigallocatechin-3-gallate (EGCG), a polyphenol found in green tea, have demonstrated its ability to protect renal cells from apoptosis and necrosis, leading to increased cell survival rates [56]. The enhancement of cell viability observed with AM treatment may be attributed to its antioxidative and anti-apoptotic mechanisms, including reducing oxidative stress, stabilizing mitochondrial function, and modulating apoptotic signaling pathways.

Beyond the primary outcomes of serum creatinine and cell viability, this meta-analysis highlights the significant impact of AM on secondary outcomes related to oxidative stress: MDA, GSH, and ROS. The observed reduction in MDA levels following AM treatment underscores its efficacy in mitigating lipid peroxidation, a hallmark of oxidative damage in AKI [45,46,48,49]. This aligns with previous studies demonstrating the antioxidant potential of AM, such as that by Zainudin et al. (2019), who showed that AM reduces lipid peroxidation and protects against oxidative stress-induced cell death in cardiomyocytes [57]. Furthermore, our analysis revealed a significant increase in GSH levels, reflecting AM’s capacity to enhance endogenous antioxidant defenses [47,49]. GSH, a crucial tripeptide antioxidant, plays a vital role in detoxifying ROS and maintaining cellular redox balance. These findings align with a study reporting that AM enhances GSH synthesis, reduces hepatic MDA, and protects against lipopolysaccharide/D-galactosamine (LPS/D-GalN)-induced acute liver failure [58]. Additionally, our analysis reveals that AM significantly reduces ROS levels, further confirming its antioxidant properties [47,49]. ROS, including superoxide radicals and hydrogen peroxide, are major contributors to oxidative stress and cellular damage in AKI. This potent antioxidant effect is corroborated by a prior investigation, which demonstrated that AM elevates glutathione (GSH) while concurrently diminishing both total and mitochondrial ROS in a rat model of high fat-diet induced hepatic steatosis [59]. The modulation of these secondary outcomes by AM likely contributes to its renoprotective effects, as oxidative stress is a central player in the pathogenesis of AKI.

The nephroprotective effects of AM in AKI models observed in this systematic review are multifaceted, extending beyond simple antioxidant activity. AM exhibits a notable ability to modulate both oxidative stress and inflammatory responses within the kidney. Emerging evidence supports the hypothesis that AM activates the Nrf2 pathway, thereby promoting antioxidant and cytoprotective gene expression AKI models [60]. Though direct measurements such as Nrf2 nuclear translocation or ARE-binding assays were not reported in the reviewed AKI studies, the observed biochemical and molecular changes strongly indicate Nrf2 pathway engagement. AM induced elevated levels of key antioxidant enzymes such as GPx, GR, and SOD, as well as increased GSH in renal tissues, which are canonical downstream targets of Nrf2 responsive to oxidative stress [11,45,61]. AM effectively suppressed oxidative stress, as evidenced by reductions in markers such as MDA, ROS, H_2_O_2_, protein carbonyls, 4-HNE, and 3-NT. These changes are consistent with enhanced Nrf2 transcriptional activity, which orchestrates cellular redox homeostasis [46,62]. Other studies outside the kidney context bolster the mechanistic plausibility in retinal pigment epithelial cells and murine retinal models, AM induced nuclear accumulation of Nrf2 and elevated expression of HO-1, confirming its capacity to activate Nrf2-driven cytoprotective pathways [63]. Extensive research in renal physiology underscores Nrf2’s pivotal role in mitigating oxidative and inflammatory damage in AKI. Pharmacological or genetic stimulation of Nrf2 has been shown to attenuate tubular injury, fibrosis, and functional decline in various kidney injury models, highlighting Nrf2 as a viable therapeutic target [62,64]. Collectively, these findings suggest that activation of the Nrf2/ARE signaling pathway likely mediates, at least in part, AM’s antioxidant, anti-inflammatory, and anti-apoptotic effects in AKI. These effects are further complemented by the observed decrease in pro-inflammatory cytokines TNF-α and IL-6, suggesting that AM actively suppresses the inflammatory cascade, possibly through inhibiting the NF-κB pathway, which contributes to AKI pathogenesis [12,45]. Decreased TNF-α and IL-6, potentially via NF-κB inhibition, suppress inflammation, similar to effects seen with erythropoietin (EPO) [65], and curcumin [52], culminating in improved renal outcomes. Furthermore, AM’s influence extends to apoptotic pathways; the included studies demonstrate that AM reduces caspase activation, modulates Bcl-2 family proteins, and suppresses p53 upregulation, reducing key apoptotic markers and further promoting cell survival [47,49]. These mechanisms directly correlate with our meta-analysis findings of reduced MDA and ROS levels, further substantiating AM’s role in alleviating oxidative stress and inflammation in AKI.

The subgroup and sensitivity analyses performed in this meta-analysis shed further light on the nuances of AM’s nephroprotective effects. The subgroup analysis, stratifying in vitro studies by cell type (HEK296 vs. LLC-PK1), revealed a statistically significant variation in response to AM. This suggests that the cellular context and specific mechanisms of action may differ depending on the renal cell type, potentially due to variations in cellular metabolism, signaling pathways, or antioxidant capacity. Further research is needed to elucidate these cell-specific effects and to determine whether AM’s efficacy is influenced by the type of renal cells primarily affected in different AKI etiologies. The sensitivity analysis, conducted on serum creatinine levels, highlighted a dose-dependent relationship with AM’s efficacy. While the overall trend indicated a reduction in creatinine levels, the magnitude of the effect varied depending on the dose administered. This finding underscores the importance of dose optimization in future studies to identify the most effective therapeutic range for AM in treatment of AKI. The observed variability also suggests that factors such as the route of administration, the timing of treatment initiation, and the severity of AKI may influence AM’s efficacy, necessitating further investigation into these potential moderating factors. Due to the limited number of in vivo studies, subgroup analysis and formal assessment of publication bias were not feasible. However, in vitro subgroup analysis revealed cell-specific responses to AM, with HEK296 cells exhibiting more consistent viability increases than LLC-PK1 cells, suggesting potential differential cellular mechanisms. Sensitivity analysis indicated a dose-dependent effect on creatinine reduction, potentially favoring lower dosages. For cell viability, AM consistently enhanced viability across different dosages, albeit with significant heterogeneity, suggesting the presence of unmeasured moderating factors. The inability to formally evaluate publication bias due to the limited sample size highlights the necessity for further investigations to validate these findings and address potential sources of bias.

Beyond preclinical models, several human studies of *Garcinia mangostana*-derived preparations provide supportive, although indirect, evidence for the translational relevance of our findings. In a randomized, double-blind, placebo-controlled trial in healthy adults, daily consumption of a mangosteen-based beverage for 30 days significantly increased plasma antioxidant capacity and reduced C-reactive protein (CRP), without affecting liver enzymes or serum creatinine, suggesting favorable modulation of systemic oxidative and inflammatory status with preserved hepatic and renal safety [66]. Similarly, a pilot randomized dose-finding study in obese individuals with elevated high-sensitivity C-reactive protein (CRP) reported a greater reduction in C-reactive protein with a high-dose mangosteen juice blend compared with placebo, again without clinically relevant safety concerns [67]. Pharmacokinetic studies further demonstrate that xanthones, including alpha-mangostin, are absorbed and detectable in human serum and urine after ingestion of mangosteen juice, confirming systemic exposure to these bioactive compounds [68]. Although none of these clinical trials specifically evaluated AKI or renal outcomes as primary endpoints, they collectively support the biological plausibility, systemic activity, and short-term safety of mangosteen xanthones in humans. This body of evidence underscores the need for carefully designed early-phase clinical studies to determine whether the nephroprotective effects observed in experimental AKI models can be translated into meaningful kidney protection in at-risk patient populations.

Several limitations should be taken into account when interpreting the findings of this study. First, the relatively small number of included studies, particularly in vivo, limits the statistical power for subgroup analyses and precludes formal assessment of publication bias. Second, the existing studies exhibit a limited diversity in AKI induction methods, being restricted to glycerol and cisplatin models in vivo and cisplatin-induced injury in vitro; this prevents a thorough assessment of AM’s consistency across diverse AKI etiologies. Third, the exclusive use of the intraperitoneal route in existing studies restricts our ability to evaluate the impact of variations in administration routes. Fourth, significant heterogeneity was observed across several analyses, reflecting variability in study designs, dosages, and outcome measures. This heterogeneity complicates the interpretation of pooled estimates and limits generalizability. Fifth, we found it challenging to definitively confirm a dose–response relationship, given inconsistencies in dosing protocols and the limited exploration of dose ranges. Sixth, as the included studies are predominantly preclinical, using animal models or cell lines, caution is warranted when extrapolating to the complexities of human AKI. Seventh, mechanistic exploration remains incomplete; while antioxidant and anti-inflammatory effects suggest involvement of Nrf2 and NF-κB pathways, none of the included studies directly assessed Nrf2 nuclear translocation, ARE-binding activity, or NF-κB activation/inhibition. This gap limits our ability to confirm pathway-specific actions of AM and underscores the need for molecular assays in future research. Finally, our restriction to English-language publications may have excluded relevant studies published in other languages.

In summary, this systematic review and meta-analysis provides compelling evidence for AM’s nephroprotective effects in preclinical AKI models. AM consistently improves renal function, enhances cell viability, and modulates oxidative stress, apoptosis, and inflammatory markers through antioxidant, anti-inflammatory, and anti-apoptotic mechanisms. Subgroup analyses reveal that cellular context and specific mechanisms of action may differ depending on the renal cell type, and sensitivity analyses highlighted a dose-dependent relationship with AM’s efficacy. However, the study is limited by the number of included studies, observed heterogeneity, the preclinical nature of the research, and the restriction to English-language publications. Therefore, while this review highlights AM’s therapeutic potential in mitigating kidney injury, further research is needed to address these limitations and fully elucidate AM’s therapeutic potential for human use.

## 5. Conclusions

In conclusion, this systematic review and meta-analysis, based on studies rigorously assessed for quality and deemed reliable, provides evidence that AM exhibits nephroprotective effects in preclinical models of AKI. AM consistently improves renal function, enhances cell viability, and modulates oxidative stress, apoptotic markers, and inflammatory markers. However, further research is needed to address limitations and fully elucidate AM’s therapeutic potential for human use.

## Figures and Tables

**Table 1:** PICO framework outlining the key elements of the included preclinical studies investigating the effects of alpha-mangostin (AM) on acute kidney injury (AKI).

PICO Element	Description
Population (P)	Animals with experimentally induced AKI (e.g., nephrotoxic agents, ischemia–reperfusion, sepsis) and renal cell lines subjected to AKI-relevant injury (oxidative stress, nephrotoxins)
Intervention (I)	Administration of purified alpha-mangostin
Comparator (C)	Vehicle-treated or untreated control groups
Outcomes (O)	Primary: Serum creatinine, blood urea nitrogen (BUN), cell viability Secondary: Oxidative stress markers, inflammatory cytokines, apoptosis indicators, histopathological findings
Study design (S)	Preclinical in vivo and in vitro studies with a control group

**Table 2:** Characteristics of included studies evaluating the effects of alpha-mangostin (AM) in preclinical models of acute kidney injury (AKI).

Study ID	Author(s)	Country	Species/Cell Line	AKI Model	Sample Size (Per Group)	Duration of Intervention/Treatment	Route of Administration	Dose of Alpha-Mangostin	Control Group	Primary Outcome	Secondary Outcomes
1	Eltahir et al., 2023 [45]	Egypt	Male Sprague Dawley rats	Glycerol-induced AKI (50% glycerol in saline, 8 mL/kg)	10	3 days	Intraperitoneal injection	175 mg/kg	Only treated with glycerol and saline	Serum creatinine	MDA
2	Pérez-Rojas et al., 2009 [46]	United States	Male Wistar rats	Cisplatin induced AKI (7.5 mg/kg)	6–8	6, 10 days	Intraperitoneal injection	12.5, 25, 50, 100, 200 mg/kg	Only treated with Cisplatin and saline	Serum creatinine	MDA
3	Sanchez-Perez et al., 2010 [47]	United States	LLC-PK1 renal epithelial cells	Cisplatin induced AKI (100 µM)	3	1 day	Culture medium treatment	1, 2.5, 5, 7.5, 10 µM	Only treated with Cisplatin	Cell viability	GSH, ROS
4	Reyes-Fermín et al., 2019 [48]	United States	LLC-PK1 renal epithelial cells	Cisplatin induced AKI (30 µM)	5–7	1 day	Culture medium treatment	1, 2, 3, 4, 5 µM	Only treated with Cisplatin	Cell viability	MDA
5	Li et al., 2020 [49]	China	HEK293 epithelial cells	Cisplatin induced AKI (20 µM)	NR	1 day	Culture medium treatment	10, 20, 40 µM	Only treated with Cisplatin	Cell viability	GSH, MDA, ROS

AKI, acute kidney injury; NR, not reported; BUN, blood urea nitrogen; GPx, glutathione peroxidase; SOD, superoxide dismutase; MDA, malondialdehyde; GSH, reduced glutathione; TNF-α, tumor necrosis factor-alpha; IL-6, interleukin-6; ROS, reactive oxygen species.

**Table 3:** Quality assessment of 5 studies using Toxicological Data Reliability Assessment Tool (ToxRTool).

Study (Author/Year)	Criteria Group I	Criteria Group II	Criteria Group III	Criteria Group IV	Criteria Group V	Overall Score	Initial Category	Revised Category
Eltahir et al., 2023 [45]	2	5	7	2	2	18	I	I
Pérez-Rojas et al., 2009 [46]	3	5	7	3	1	20	I	I
Sanchez-Perez et al., 2010 [47]	4	3	6	3	2	18	I	I
Reyes-Fermín et al., 2019 [48]	3	3	6	3	2	17	I	I
Li et al., 2020 [49]	3	3	6	3	2	17	I	I

Criteria group I, identification of the test substance; criteria group II, characterization of the test system; criteria group III, description of the study design; criteria group IV, documentation of study results; criteria group V, plausibility of the study design and findings; categories I, reliable without restrictions; categories II, reliable with restrictions, categories III, unreliable.

**Table 4:** Summary of alpha-mangostin’s protective effects in renal injury models, highlighting key biomarkers and outcomes. ↑ indicates increase, ↓ indicates decrease.

Study	Model Type	Other Oxidative Stress Markers	Inflammatory Markers	Apoptosis Markers	Histopathological Findings	Key Findings Related to Protection
Eltahir et al., 2023 [45]	In vivo	GPx ↑, GRs ↑, SOD ↑	TNF-α ↓, IL-6 ↓	N/A	Improved renal histological features, alleviated renal edema	Alleviated renal damage in glycerol-induced AKI.
Pérez-Rojas et al., 2009 [46]	In vivo	Renal H_2_O_2_ ↓, protein carbonyls ↓, GSH ↑, 4-HNE ↓, 3-NT ↓	N/A	N/A	Attenuate renal tubular injury at both structural and functional levels	Attenuated renal dysfunction, structural damage, oxidative/nitrosative stress, and inflammatory markers in cisplatin-induced nephrotoxicity in rats.
Sánchez-Pérez et al., 2010 [47]	In vitro	N/A	N/A	p53 ↑	N/A	Protected renal tubular cells by blocking cisplatin-induced apoptosis through ROS generation and p53 signaling.
Reyes-Fermin et al., 2019 [48]	In vitro	N/A	N/A	N/A	N/A	Exerted an anti-apoptotic effect.
Li et al., 2020 [49]	In vitro	N/A	N/A	Caspase 3 ↓, caspase 9 ↓, PARP cleavage ↓, Bad ↓, Bax↓, Bcl-xl ↑, Bcl-2 ↑	N/A	Reversed cisplatin-induced toxicity by inhibiting ROS and modulating PI3K/Akt and JNK.

GPx, glutathione peroxidase; GRs, glutathione reductase; SOD, superoxide; TNF- α, tumor necrosis factor-alpha; IL-6, interleukun-6; H_2_O_2_, hydrogen peroxide; GSH, glutathione; 4-HNE, 4-hydroxy-2-noneal; 3-NT, 3-nitrotyrosine; N/A, not applicable, ROS, reactive oxygen species, PARP, poly (ADP-ribose) polymerase; Bax, Bcl-2-associated X protein; Bad, Bcl-2-associated death promoter; Bcl-xl, B-cell lymphoma-extra large; Bcl-2, B-cell lymphoma 2; PI3K, phosphatidylinositol 3-kinase; Akt, protein kinase B; JNK, c-Jun N-terminal kinase; AKI, acute kidney injury.

## Data Availability

The original contributions presented in this study are included in the article/Supplementary Material. Further inquiries can be directed to the corresponding author.

## Supplementary Materials

The following supporting information can be downloaded at: https://www.mdpi.com/article/10.3390/antiox14111374/s1, Table S1: PRISMA checklist; Table S2: Search strategy in PubMed and Scopus; Table S3: Search strategy in Embase; Table S4: Search strategy in Web of Science.

## Abbreviations

The following abbreviations are used in this manuscript:

3-NT	3-Nitrotyrosine
4-HNE	4-Hydroxynonenal
AKI	Acute kidney injury
AM	Alpha-mangostin
BUN	Blood urea nitrogen
CRP	C-reactive protein
CI	Confidence interval
EPO	Erythropoietin
GPx	Glutathione peroxidase
GR	Glutathione reductase
GSH	Glutathione
H2O2	Hydrogen peroxide
I2	I-squared (a measure of heterogeneity)
IL-6	Interleukin-6
MD	Mean difference
MDA	Malondialdehyde
NF-κB	Nuclear factor kappa B
OSF	Open Science Framework
PARP	Poly (ADP-ribose) polymerase
PRISMA	Preferred Reporting Items for Systematic Reviews and Meta-Analyses
ROS	Reactive oxygen species
SD	Standard deviation
SEM	Standard error of mean
SOD	Superoxide dismutase
TNF-α	Tumor necrosis factor-alpha
ToxRTool	Toxicological data reliability assessment tool
