# Supplementary material for: The Nephroprotective Effects of Alpha-Mangostin for Acute Kidney Injury: A Systematic Review and Meta-Analysis
Source: Antioxidants (Basel). 2025 Nov 19;14(11):1374. doi: 10.3390/antiox14111374 (PMC12649582; doi:10.3390/antiox14111374)
Supplement: Supplementary file 1 [file antioxidants-14-01374-s001.zip › antioxidants-3981542-supplementary.pdf]

The Nephroprotective Effects of Alpha-Mangostin for Acute Kidney Injury: A Systematic Review and Meta-Analysis

Moragot Chatatikun [1,2], Aman Tedasen [1,2], Ratana Netphakdee [1], Jitbanjong Tangpong [1,2], Phichayut Phinyo [3,4], Pakpoom Wongyikul [3,4], Fumitaka Kawakami [5,6], Makoto Kubo [6,7], Motoki Imai [7,8], Wiyada Kwanhian Klangbud [9], Atthaphong Phongphithakchai [10,*]

1 Department of Medical Technology, School of Allied Health Sciences, Walailak University, Nakhon Si Thammarat 80160, Thailand
2 Research Excellence Center for Innovation and Health Products (RECIHP), Walailak University, Nakhon Si Thammarat 80160, Thailand
3 Center for Clinical Epidemiology and Clinical Statistics, Faculty of Medicine, Chiang Mai University, Chiang Mai 50200, Thailand
4 Department of Biomedical Informatics and Clinical Epidemiology (BioCE), Faculty of Medicine, Chiang Mai University, Chiang Mai 50200, Thailand
5 Department of Regulation Biochemistry, Kitasato University Graduate School of Medical Sciences, Sagamihara, Japan
6 Department of Environmental Microbiology, Kitasato University Graduate School of Medical Sciences, Sagamihara, Japan
7 Regenerative Medicine and Cell Design Research Facility, School of Allied Health Sciences, Kitasato, Sagamihara, Japan
8 Department of Molecular Diagnostics, School of Allied Health Sciences, Kitasato University, 1-15-1 Kitasato, Sagamihara 252-0373, Kanagawa, Japan
9 Medical Technology Program, Faculty of Science, Nakhon Phanom University, Nakhon Phanom 48000, Thailand
10 Nephrology Unit, Division of Internal Medicine, Faculty of Medicine, Prince of Songkla University, Hat Yai, Songkhla 90110, Thailand
* Correspondence: atthaphong.p@psu.ac.th

Supplementary Table S1. PRISMA checklist

Section and Topic	Item #	Checklist item	Location where item is reported
**TITLE**			
Title	1	Identify the report as a systematic review.	Page 1
**ABSTRACT**			
Abstract	2	See the PRISMA 2020 for Abstracts checklist.	Page 1-2
**INTRODUCTION**			
Rationale	3	Describe the rationale for the review in the context of existing knowledge.	Page 2-3
Objectives	4	Provide an explicit statement of the objective(s) or question(s) the review addresses.	Page 3
**METHODS**			
Eligibility criteria	5	Specify the inclusion and exclusion criteria for the review and how studies were grouped for the syntheses.	Page 4
Information sources	6	Specify all databases, registers, websites, organisations, reference lists and other sources searched or consulted to identify studies. Specify the date when each source was last searched or consulted.	Pages 3-4
Search strategy	7	Present the full search strategies for all databases, registers and websites, including any filters and limits used.	Supplementary Tables S2–S6
Selection process	8	Specify the methods used to decide whether a study met the inclusion criteria of the review, including how many reviewers screened each record and each report retrieved, whether they worked independently, and if applicable, details of automation tools used in the process.	Page 4
Data collection process	9	Specify the methods used to collect data from reports, including how many reviewers collected data from each report, whether they worked independently, any processes for obtaining or confirming data from study investigators, and if applicable, details of automation tools used in the process.	Pages 4
Data items	10a	List and define all outcomes for which data were sought. Specify whether all results that were compatible with each outcome domain in each study were sought (e.g. for all measures, time points, analyses), and if not, the methods used to decide which results to collect.	Pages 5-6
	10b	List and define all other variables for which data were sought (e.g. participant and intervention characteristics, funding sources). Describe any assumptions made about any missing or unclear information.	Pages 4-6
Study risk of bias assessment	11	Specify the methods used to assess risk of bias in the included studies, including details of the tool(s) used, how many reviewers assessed each study and whether they worked independently, and if applicable, details of automation tools used in the process.	Page 5
Effect measures	12	Specify for each outcome the effect measure(s) (e.g. risk ratio, mean difference) used in the synthesis or presentation of results.	Pages 5-6
Synthesis methods	13a	Describe the processes used to decide which studies were eligible for each synthesis (e.g. tabulating the study intervention characteristics and comparing against the planned groups for each synthesis (item #5)).	Pages 5-6
	13b	Describe any methods required to prepare the data for presentation or synthesis, such as handling of missing summary statistics, or data conversions.	Pages 5-6

Supplementary Table S1. PRISMA checklist

Section and Topic	Item #	Checklist item	Location where item is reported
	13c	Describe any methods used to tabulate or visually display results of individual studies and syntheses.	Pages 5-6
	13d	Describe any methods used to synthesize results and provide a rationale for the choice(s). If meta-analysis was performed, describe the model(s), method(s) to identify the presence and extent of statistical heterogeneity, and software package(s) used.	Pages 5-6
	13e	Describe any methods used to explore possible causes of heterogeneity among study results (e.g. subgroup analysis, meta-regression).	Pages 5-6
	13f	Describe any sensitivity analyses conducted to assess robustness of the synthesized results.	Pages 5-6
Reporting bias assessment	14	Describe any methods used to assess risk of bias due to missing results in a synthesis (arising from reporting biases).	Page 5
Certainty assessment	15	Describe any methods used to assess certainty (or confidence) in the body of evidence for an outcome.	Pages 5-6
**RESULTS**			
Study selection	16a	Describe the results of the search and selection process, from the number of records identified in the search to the number of studies included in the review, ideally using a flow diagram.	Page 5
	16b	Cite studies that might appear to meet the inclusion criteria, but which were excluded, and explain why they were excluded.	Page 5
Study characteristics	17	Cite each included study and present its characteristics.	Pages 7-8
Risk of bias in studies	18	Present assessments of risk of bias for each included study.	Page 9
Results of individual studies	19	For all outcomes, present, for each study: (a) summary statistics for each group (where appropriate) and (b) an effect estimate and its precision (e.g. confidence/credible interval), ideally using structured tables or plots.	Pages 10-17
Results of syntheses	20a	For each synthesis, briefly summarise the characteristics and risk of bias among contributing studies.	Pages 7-9
	20b	Present results of all statistical syntheses conducted. If meta-analysis was done, present for each the summary estimate and its precision (e.g. confidence/credible interval) and measures of statistical heterogeneity. If comparing groups, describe the direction of the effect.	Pages 9-17
	20c	Present results of all investigations of possible causes of heterogeneity among study results.	Pages 9-17
	20d	Present results of all sensitivity analyses conducted to assess the robustness of the synthesized results.	Pages 15-16
Reporting biases	21	Present assessments of risk of bias due to missing results (arising from reporting biases) for each synthesis assessed.	Page 9
Certainty of evidence	22	Present assessments of certainty (or confidence) in the body of evidence for each outcome assessed.	Pages 9, 17
**DISCUSSION**			

Supplementary Table S1. PRISMA checklist

Section and Topic	Item #	Checklist item	Location where item is reported
Discussion	23a	Provide a general interpretation of the results in the context of other evidence.	Pages 17-21
	23b	Discuss any limitations of the evidence included in the review.	Page 17-21
	23c	Discuss any limitations of the review processes used.	Page 20-21
	23d	Discuss implications of the results for practice, policy, and future research.	Page 20-21
**OTHER INFORMATION**			
Registration and protocol	24a	Provide registration information for the review, including register name and registration number, or state that the review was not registered.	Pages 3-4
	24b	Indicate where the review protocol can be accessed, or state that a protocol was not prepared.	Pages 3-4
	24c	Describe and explain any amendments to information provided at registration or in the protocol.	Not applicable
Support	25	Describe sources of financial or non-financial support for the review, and the role of the funders or sponsors in the review.	Page 21
Competing interests	26	Declare any competing interests of review authors.	Page 22
Availability of data, code and other materials	27	Report which of the following are publicly available and where they can be found: template data collection forms; data extracted from included studies; data used for all analyses; analytic code; any other materials used in the review.	Page 21

Supplementary Table S2. Search strategy in PubMed and Scopus.

Database	Search query	Results
PubMed	((((((((((acute kidney injury[Title/Abstract]) OR (AKI[Title/Abstract])) OR (acute renal failure[Title/Abstract])) OR (renal injury[Title/Abstract])) OR (nephrotoxicity[Title/Abstract])) OR (ischemia reperfusion injury[Title/Abstract])) OR (renal ischemia[Title/Abstract])) OR (drug-induced kidney injury[Title/Abstract])) OR (cisplatin nephrotoxicity[Title/Abstract])) OR (gentamicin nephrotoxicity[Title/Abstract])) AND (((((alpha-mangostin[Title/Abstract]) OR (α-mangostin[Title/Abstract])) OR (mangosteen extract[Title/Abstract])) OR (Garcinia mangostana[Title/Abstract])) OR (xanthone[Title/Abstract]))) AND (((((((((((animal[Title/Abstract]) OR (animal experiment[Title/Abstract])) OR (preclinical[Title/Abstract])) OR (in vivo[Title/Abstract])) OR (in vitro[Title/Abstract])) OR (cell culture[Title/Abstract])) OR (experimental study[Title/Abstract])) OR (rodent[Title/Abstract])) OR (rat[Title/Abstract])) OR (mouse[Title/Abstract])) OR (mice[Title/Abstract]))	11
Scopus	((TITLE-ABS-KEY (acute kidney injury)) OR (TITLE-ABS-KEY (AKI)) OR (TITLE-ABS-KEY (acute renal failure)) OR (TITLE-ABS-KEY (renal injury)) OR (TITLE-ABS-KEY (nephrotoxicity)) OR (TITLE-ABS-KEY (ischemia reperfusion injury)) OR (TITLE-ABS-KEY (renal ischemia)) OR (TITLE-ABS-KEY (drug-induced kidney injury)) OR (TITLE-ABS-KEY (cisplatin nephrotoxicity)) OR (TITLE-ABS-KEY (gentamicin nephrotoxicity))) AND ((TITLE-ABS-KEY (alpha-mangostin)) OR (TITLE-ABS-KEY (α-mangostin)) OR (TITLE-ABS-KEY (mangosteen extract)) OR (TITLE-ABS-KEY (Garcinia mangostana)) OR (TITLE-ABS-KEY (xanthone)))	87

Supplementary Table S3. Search strategy in Embase.

No.	Search query	Results
1	acute kidney injury.ab,ti.	80367
2	AKI.ab,ti.	52833
3	acute renal failure.ab,ti.	35940
4	renal injury.ab,ti.	22959
5	nephrotoxicity.ab,ti.	30667
6	ischemia reperfusion injury.ab,ti.	43490
7	renal ischemia.ab,ti.	7690
8	drug-induced kidney injury.ab,ti.	215
9	cisplatin nephrotoxicity.ab,ti.	832
10	gentamicin nephrotoxicity.ab,ti.	394
11	1 or 2 or 3 or 4 or 5 or 6 or 7 or 8 or 9 or 10	207650
12	alpha-mangostin.ab,ti.	819
13	mangosteen extract.ab,ti.	45
14	Garcinia mangostana.ab,ti.	861
15	xanthone.ab,ti.	2372
16	12 or 13 or 14 or 15	3414
17	11 and 16	20

Supplementary Table S4. Search strategy in Web of Science.

No.	Search query	Results
1	acute kidney injury (All Fields)	72840
2	AKI (All Fields)	45147
3	acute renal failure (All Fields)	52218
4	renal injury (All Fields)	92583
5	nephrotoxicity (All Fields)	24676
6	ischemia reperfusion injury (All Fields)	77132
7	renal ischemia (All Fields)	23170
8	drug-induced kidney injury (All Fields)	1629
9	cisplatin nephrotoxicity (All Fields)	5244
10	gentamicin nephrotoxicity (All Fields)	1814
11	#10 OR #9 OR #8 OR #7 OR #6 OR #5 OR #4 OR #3 OR #2 OR #1	256227
12	alpha-mangostin (All Fields)	1056
13	α-mangostin (All Fields)	837
14	mangosteen extract (All Fields)	637
15	Garcinia mangostana (All Fields)	1395
16	xanthone (All Fields)	5731
17	#12 OR #13 OR #14 OR #16 OR #15	6885
18	#17 AND #11	53
